# Scientific Evidences of Calorie Restriction and Intermittent Fasting for Neuroprotection in Traumatic Brain Injury Animal Models: A Review of the Literature

**DOI:** 10.3390/nu14071431

**Published:** 2022-03-30

**Authors:** Yang Xu, Zejie Liu, Shuting Xu, Chengxian Li, Manrui Li, Shuqiang Cao, Yuwen Sun, Hao Dai, Yadong Guo, Xiameng Chen, Weibo Liang

**Affiliations:** 1 West China School of Basic Medical Sciences and Forensic Medicine, Sichuan University, Chengdu 610041, China; 19944501946a@gmail.com (Y.X.); 2021151610079@stu.scu.edu.cn (S.X.); chengxianli@stu.scu.edu.cn (C.L.); 2017151611001@stu.scu.edu.cn (Y.S.); 2 Department of Forensic Pathology and Forensic Clinical Medicine, West China School of Basic Medical Sciences and Forensic Medicine, Sichuan University, Chengdu 610041, China; 18959396963@163.com (Z.L.); dai.hao22@gmail.com (H.D.); 3 Department of Forensic Genetics, West China School of Basic Medical Sciences and Forensic Medicine, Sichuan University, Chengdu 610041, China; marylee078@gmail.com (M.L.); caoshuqiang916@163.com (S.C.); 4 Department of Forensic Science, School of Basic Medical Sciences, Central South University, Changsha 410013, China; gdy82@126.com

**Keywords:** traumatic brain injury, calorie restriction, intermittent fasting, mitochondrial dysfunction, hippocampal neurogenesis, glial cell responses, neural cell plasticity, apoptosis, autophagy

## Abstract

It has widely been accepted that food restriction (FR) without malnutrition has multiple health benefits. Various calorie restriction (CR) and intermittent fasting (IF) regimens have recently been reported to exert neuroprotective effects in traumatic brain injury (TBI) through variable mechanisms. However, the evidence connecting CR or IF to neuroprotection in TBI as well as current issues remaining in this research field have yet to be reviewed in literature. The objective of our review was therefore to weigh the evidence that suggests the connection between CR/IF with recovery promotion following TBI. Medline, Google Scholar and Web of Science were searched from inception to 25 February 2022. An overwhelming number of results generated suggest that several types of CR/IF play a promising role in promoting post-TBI recovery. This recovery is believed to be achieved by alleviating mitochondrial dysfunction, promoting hippocampal neurogenesis, inhibiting glial cell responses, shaping neural cell plasticity, as well as targeting apoptosis and autophagy. Further, we represent our views on the current issues and provide thoughts on the future direction of this research field.

## 1. Introduction

Since ancient times, worldwide religions have advocated for food restriction (FR) due to its physical and psychological benefits [1,2,3]. However, the health benefits owing to FR have only in recent years been supported by scientific evidence. Calorie restriction (CR) is a commonly used FR strategy that restricts everyday energy intake without incurring malnutrition [4]. In experimental animal models, appropriate CR has been shown to have benefits including elongation of life span [5], promotion of weight loss [6,7,8], suppression of inflammation [9,10,11], cardiovascular disease risk reduction [12,13,14], and cancer prevention [2,15]. Notwithstanding these benefits, there are still concerns when applying CR, such as poor CR compliance [16]. Researchers have therefore been looking for alternative FR regimens that can provide similar benefits. One of these regimens is intermittent fasting (IF), and it has recently become a popular trend and lifestyle [17]. IF refers to cycles of fasting and intermittent feeding window over a given time schedule [2,16,18]. There are various time-scheduled IF methods, the following three are the most common approaches: periodic fasting (PE), time-restricted feeding (TRF) and alternate-day fasting (ADF) [16]. The IF ultimately triggers a process called “the metabolic switch”, which shifts the metabolism from glycogenolysis to the mobilization of fat via fatty acid oxidation and ketogenesis, resulting in related biochemical pathway changes [1,19,20]. It is widely accepted that these changes under IF positively contributes to human health in multiple areas [21,22,23,24]. Recent studies in the field of neuroscience have found scientific evidence that IF exerts protective effects against multiple neurological diseases and disorders, including Alzheimer’s disease (AD) [25,26], Parkinson’s disease (PD) [27,28], multiple sclerosis (MS) [29,30], epilepsy [31,32], ischemic stroke [33,34,35], and depression [36,37,38].

Traumatic brain injury (TBI), a temporary or permanent disruption of normal brain function due to damage incurred by external forces [39], is a major burden on those effected [40,41,42]. After the initial brain trauma, the secondary injury process spreads via a complex sequence of events in the pathogenesis of TBI [43,44,45]. Therapeutics targeting the development of the secondary injuries play a key role in limiting disabilities incurred from TBI while achieving unsatisfactory clinical outcomes [46,47]. To improve the quality of life for patients suffering from TBI, the search for more effective interventions for TBI has to continue. Our study recently found that a 1-month IF regimen prior to TBI significantly improved cognitive function following the insult, in which Npy (Neuropeptide Y)-induced neurogenesis made a contribution [48]. In addition, several studies have shown that multiple FR regimens, mainly CR and IF, exert positive effects on the development of TBI via various mechanisms [19,49,50]. However, the scientific evidence bridging CR/IF with neuroprotection in TBI has not been systematically reviewed in previous literature. Additionally, concerns regarding this research field remain elusive. To this end, here we have retrospectively reviewed the recent studies on CR/IF in the TBI, summarized the current issues, and presented our consideration of future directions in this research field.

## 2. Neuroprotective Effects of CR and IF in TBI

### 2.1. Alleviating Mitochondrial Dysfunction

The preservation of normal mitochondrial function is critical for inhibiting the deterioration of secondary TBI injury [51,52,53]. Davis et al. reported that acute fasting (for a single 24 h) in adult male rats, after receiving moderate cortical control impact (CCI), significantly increased tissue-sparing postinjury [54]. The underlying mechanism is suggested to be reduced mitochondrial damage, as indicated by lower levels of mitochondrial reactive oxygen species (ROS) production, calcium loading, lipid peroxidation and protein carbonyls [55]. Additionally, administration and maintenance of serum d-bHB at a level similar to the fasting state also alleviated mitochondrial dysfunction and increased tissue sparing after moderate CCI, indicating the neuroprotective role of fasting was achieved by elevated d-bHB level. Notably, a higher dose of d-bHB did not present similar neuroprotective effects, suggesting a dose-dependent effect on the efficacy of serum ketones in TBI [54]. Studies on the effects of CR on mitochondrial protection following TBI are limited. However, since CR usually induces ketone production, the study of ketogenic diet (KD) may provide some clues. KD was recently found to reduce oxidative stress following CCI, while inducing the levels of antioxidants, superoxide dismutase (SOD1/2), and NAD(P)H dehydrogenase, thereby improving mitochondrial respiratory complex activity [56]. Whether this effect can be achieved by fasting or simply calorie restriction still needs to be explored. Additionally, several variables must be considered; for instance, current studies only involve male animal models, so whether food restriction has a similar effect on the female remains to be determined. Moreover, age should also be taken into consideration. Furthermore, although Davis et al. reported that a prolonged fasting (48 h) did not show profound neuroprotection, further investigation would be required to determine whether IF or daily calorie restriction could produce the mitochondrial protection. Finally, CR alters the level of numerous factors [57,58,59,60,61] besides d-bHB, but other factors contributing to the improved mitochondrial function also requires further investigation. The screening of overlapping differentially expressed genes (DEGs) by CR-induced and brain injury-induced transcriptional changes may be valuable.

### 2.2. Promoting Hippocampal Neurogenesis

Hippocampal dysfunction is a major pathological aspect of TBI that generally affects patients’ spatial learning and memory [62,63,64,65]. We recently found that a 1-month IF regimen other than acute fasting prior to CCI significantly enhanced the proliferation of neural stem cells (NSCs) in the subgranular zone (SGZ) of the hippocampus and improved cognitive function postinjury [48]. A subsequent loss of function study demonstrated that the neurogenesis-promoting effect of IF following TBI was achieved by increasing the neuronal NPY expression in the hippocampus [48]. Our study, contrary to that of Davis, suggests the health benefits of an IF regimen in TBI and its underlying mechanisms. Davis’s conclusion demonstrated that a single 24 h period of fasting after moderate CCI exhibited neuroprotection postinjury [54]. The cause of this contradiction may be due to the differences in species, but this requires further investigation.

To the best of our knowledge, no other study has reported the effects of IF or other types of CR on hippocampal neurogenesis after TBI. In ischemic brain injury, a three-month IF regimen prior to injury was found to limit both the proliferation of basal cells and cell death in the subgranular region of the hippocampus and SVZ—two regions of sustained neurogenesis in the adult brain [66]. However, this regimen promoted cell proliferation in the dentate gyrus of intact mice. These results raised the following questions: (1) Does the 1-month IF regimen promote or inhibit hippocampal basal cell proliferation following TBI? (2) If the fasting regimen was prolonged to 3 months, would there be any difference in the behavior of NSCs in the SGZ as we reported [48]?

Other issues must also be addressed. For instance, in a noninjury study, IF was reported to promote neurogenesis by stimulating ketogenic effects [67]. In our TBI study, the 1-month IF regimen was also found to be ketogenic; thus, whether the increased expression of Npy and enhanced neurogenesis under IF were achieved through the ketogenic effect remains to be explored. Further, some noninjury studies found that factors such as brain-derived neurotrophic factor (BNDF) and neurotrophic molecule-3 (NT-3) also participate in the IF-induced neurogenesis or differentiation of neurons in the hippocampus [68,69]. Therefore, investigations targeting other pathways or factors in the IF-induced benefits following TBI are valuable.

### 2.3. Inhibiting Glial Cell Responses

It is generally accepted that glial cell activation following brain injury plays a critical role in the progression of secondary injury following the primary insult [70,71,72,73,74], such as triggering the neuroinflammatory responses [75,76,77,78,79]. Adult male mice on a three-month CR regimen (50% of a normal daily diet) prior to TBI were found to have significantly reduced microglia activation one month after injury. As a result, the release of proinflammatory cytokines (e.g., TNF-α) was profoundly inhibited, thus reducing the neuroinflammation and ameliorating neurological damage after TBI [80]. Consistently, another non-TBI study in rats also demonstrated the anti-inflammatory properties of CR, which reduced microglia activation in the hypothalamic arcuate nucleus (ARC) [81].

For astrocytes [82], a 3-month 50% CR prior to cortical puncture has been shown to lower the number of reactive astrocytes in the injured area [83]. Consistently, in another rat model study, a 1-month CR regimen following moderate TBI demonstrated the reduction of GFAP-positive cells in the hippocampal CA3 region, and improved cognitive dysfunction [84]. Notably, a ketogenic diet following moderate TBI was also found to prevent neuroinflammation and inhibit astrocytes activation in male mice [50]. Therefore, suppression of astrocyte activation by CR may involve the production of ketone bodies. In addition, IF has been shown to reduce astrocyte activation in other types of brain injury, s86uch as the kainic acid-induced brain injury [85]. In summary, these results suggest that the CR, either prior to or post injury, inhibits astrocyte activation and glia scar formation following TBI. These results, in future medicine, have the potential to guide clinical intervention in the progression of secondary brain injury [86,87,88].

### 2.4. Shaping Neural Cell Plasticity

In the pathogenesis of TBI, neural cell plasticity (including synaptogenesis, alterations of neural cell structure, and change in growth factor signaling, etc.) remodels to promote functional recovery [89,90,91,92,93]. Earlier, Nataša et al. reported that male rats maintained on a 3-month 50% food restriction (FR) prior to TBI exhibited quantitative changes in synaptophysin (SYP), growth-associated protein 43 (GAP-43), and glial fibrillary acidic protein (GFAP)—markers of neuronal and glial plasticity, respectively [83]. FR profoundly raised GAP-43 and SYP expression in the cortex surrounding the lesion, indicating increased axonal branching and synapses [83]. In ad libitum (AL) rats, reactive astrocytes have been observed without immunoactivity for GAP-43 or SYP [83]. These results suggest that the FR promotes recovery by enhancing neuronal plasticity while inhibiting reactive astrogliosis. In a noninjury study, a daily IF regimen (22 h fasting per day) for 3 weeks significantly increased mushroom dendritic spine density and hippocampal expression of BDNF, suggesting a change in the dendritic spine remodeling (2). Altogether, these data point to the conclusion that a relatively long period of FR may improve cognitive function following TBI by shaping neural cell plasticity, in which multiple pathways and factors are involved. Whether short-term or acute calorie restriction has similar benefits still remains to be determined. In addition, the effect of IF on female animal models remain elusive and need to be investigated in future studies.

### 2.5. Targeting Apoptosis and Autophagy

Studies have shown that apoptosis and autophagy play detrimental roles in the pathogenesis of TBI [94,95,96,97]. By targeting apoptosis or the activity of autophagy, progression of TBI can possibly be attenuated [94,95]. Earlier, Natasa et al. demonstrated that a 50% CR regimen lasting 3 months, prior to cortical stab injury (CSI), suppressed caspase-3 expression in the ipsilateral cortex, which attenuated the secondary injury after the primary insult (3). The same group later reported that the 50% dietary restriction (DR) for 3 months before TBI affected the intrinsic apoptotic pathway [98]. The DR prevented the increase of the proapoptotic gene Bax while enhanced the expression of antiapoptotic genes Bcl-2 and Bcl-xl in the ipsilateral cortex post injury (4).

Additionally, CR has been reported to affect autophagy. In one study, rats were fed a 70% CR diet for a period of 3 months after mild TBI (mTBI) was induced, the findings revealed decreased the mammalian target of rapamycin (mTOR) activity [84], which is responsible for the inhibition of autophagy. In the same study, enhanced LC3B expression in the hippocampus [84], which promotes autophagy, was also noted. In addition, the postinjury cognitive dysfunction was ameliorated under the CR. These results suggest that the CR after mTBI improves cognition by enhancing hippocampal autophagy.

The above studies support the idea that a variable degree of calorie cut promotes neural cell survival in both the cortex and hippocampus, via different pathways. However, investigations on female animal models are still lacking. A more systematic comparative study on the effect of different ratios and durations of energy cuts in preventing cell death or promoting cell survival in different brain regions is still needed. More reputable loss-of-function studies could be helpful in bridging these mechanisms with improved cognition or tissue spares after TBI.

## 3. Current Issues and Perspectives

Although various types of CR have been shown to bring health benefits via multiple mechanisms in TBI, there are still several issues to be addressed. (1) The IF and simply CR must be clearly distinguished. In simply CR studies, as the everyday insatiety promotes cravings for food, pellets are consumed in a short time once provided. Therefore, the experimental subjects were left without food for the time remaining, consequently allowing them to enter the IF state. In some IF studies, whether the neuroprotective effect is achieved via a specific fasting regimen or simply by energy restriction should also be determined. To clearly distinguish between these two conditions, we suggest that food pellets be provided several times per day in simply CR studies, to avoid long fasting intervals. (2) As the recovery process initiates immediately after TBI, but acute CR was not able to cause significant improvement in the recovery within a very short time, thus previous studies usually planned the long-term CR regimen before injury. This raises the question of whether the therapeutic relevance of such an approach might be zero. Investigating the neuroprotective mechanisms of long-term CR and thence screening for therapeutic targets that mimic the benefits of CR may be helpful. In future research, the post-traumatic CR regimen may need to be modified to test for a therapeutic effect. (3) Female animal models are usually not involved in previous studies. Due to the roller-coaster ride of the sex hormone in menstrual cycle, female subjects usually exhibit huge variability in response to CR. Keeping the other variables consistent and largely increasing the N number in the experiment could potentially ameliorate this issue. (4) In our study, we found that when the daily energy cut was more than 30% over a prolonged period of 10 days, the mice become thin, weak, and inactive. Thus, h18ealth conditions, especially the risk of malnutrition, should be carefully monitored and reported in CR studies. (5) Circulating metabolites, such as blood glucose, β-hydroxybutyric acid, and insulin, greatly change during CR; however, this has yet to be extensively studied in TBI cases. Some of them might act as the mediators connecting the CR with its neuroprotective effect. Furthermore, the levels of these metabolites can easily be changed through clinical interventions. Thus, we suggest further investigations of the role of these metabolites in future TBI studies.

## Data Availability

Not applicable.

## References

[1] Vasim I., Majeed C.N., DeBoer M.D. (2022). Intermittent Fasting and Metabolic Health. Nutrients.

[2] Clifton K.K., Ma C.X., Fontana L., Peterson L.L. (2021). Intermittent fasting in the prevention and treatment of cancer. CA Cancer J. Clin..

[3] Tootee A., Larijani B. (2020). Ramadan fasting during COVID-19 pandemic. J. Diabetes Metab. Disord..

[4] Flanagan E.W., Most J., Mey J.T., Redman L.M. (2020). Calorie Restriction and Aging in Humans. Annu. Rev. Nutr..

[5] Cerqueira F.M., Cunha F.M., Laurindo F.R., Kowaltowski A.J. (2012). Calorie restriction increases cerebral mitochondrial respiratory capacity in a NO*-mediated mechanism: Impact on neuronal survival. Free Radic. Biol. Med..

[6] Korybalska K., Swora-Cwynar E., Luczak J., Kanikowska A., Czepulis N., Rutkowski R., Breborowicz A., Grzymislawski M., Witowski J. (2016). Association of endothelial proliferation with the magnitude of weight loss during calorie restriction. Angiogenesis.

[7] Magkos F. (2022). Is calorie restriction beneficial for normal-weight individuals? A narrative review of the effects of weight loss in the presence and absence of obesity. Nutr. Rev..

[8] Sun J., Xu N., Lin N., Wu P., Yuan K., An S., Zhang Z., Ruan Y., Zhang Y., Xu G. (2019). 317-LB: Optimal Weight Loss Effect of Short-Term Low Carbohydrate Diet with Calorie Restriction on Overweight/Obese Subjects in South China—A Multicenter Randomized Controlled Trial. Diabetes.

[9] Park C.Y., Park S., Kim M.S., Kim H.K., Han S.N. (2017). Effects of mild calorie restriction on lipid metabolism and inflammation in liver and adipose tissue. Biochem. Biophys. Res. Commun..

[10] Kim D.H., Lee E.K., Park M.H., Kim B.-C., Chung K.W., Yu B.P., Chung H.Y. (2015). Anti-inflammatory Action of Calorie Restriction Underlies the Retardation of Aging and Age-Related Diseases. Nutr. Exerc. Epigenetics Ageing Interv..

[11] Hoong C.W.S., Chua M.W.J. (2021). SGLT2 inhibitors as calorie restriction-mimetics: Insights on longevity pathways and age-related diseases. Endocrinology.

[12] Giacomello E., Toniolo L. (2021). The Potential of Calorie Restriction and Calorie Restriction Mimetics in Delaying Aging: Focus on Experimental Models. Nutrients.

[13] Silveira É.A., Noll P.R.e.S., Mohammadifard N., Rodrigues A.P.S., Sarrafzadegan N., de Oliveira C. (2021). Which Diets Are Effective in Reducing Cardiovascular and Cancer Risk in Women with Obesity? An Integrative Review. Nutrients.

[14] Perry C.A., Gadde K.M. (2022). The Role of Calorie Restriction in the Prevention of Cardiovascular Disease. Curr. Atheroscler. Rep..

[15] Vidoni C., Ferraresi A., Esposito A., Maheshwari C., Dhanasekaran D.N., Mollace V., Isidoro C. (2021). Calorie Restriction for Cancer Prevention and Therapy: Mechanisms, Expectations, and Efficacy. J. Cancer Prev..

[16] Duregon E., Pomatto-Watson L., Bernier M., Price N.L., de Cabo R. (2021). Intermittent fasting: From calories to time restriction. Geroscience.

[17] Mohiuddin A.K. (2019). Intermittent Fasting and Adding More days to Life. J. Gastroenterol. Hepatol. Res..

[18] Fallows E., Mckenzie H. (2021). Intermittent Fasting: A Health Panacea or Just Calorie Restriction? A Prescription for Healthy Living.

[19] Gudden J., Arias Vasquez A., Bloemendaal M. (2021). The Effects of Intermittent Fasting on Brain and Cognitive Function. Nutrients.

[20] Rajpal A., Ismail-Beigi F. (2020). Intermittent Fasting and “Metabolic Switch”: Effects on Metabolic Syndrome, Pre-diabetes and Type 2 Diabetes Mellitus. Diabetes Obes. Metab..

[21] Varady K. (2021). Intermittent fasting is gaining interest fast. Nat. Rev. Mol. Cell Biol..

[22] Matiashova L., Shanker A., Isayeva G. (2021). The effect of intermittent fasting on mortality in patients with type 2 diabetes and metabolic disease with high cardiovascular risk: A systematic review. Clin. Diabetol..

[23] Dwaib H.S., AlZaim I., Eid A.H., Obeid O., El-Yazbi A.F. (2021). Modulatory Effect of Intermittent Fasting on Adipose Tissue Inflammation: Amelioration of Cardiovascular Dysfunction in Early Metabolic Impairment. Front. Pharmacol..

[24] Vemuganti R., Arumugam T.V. (2020). Molecular Mechanisms of Intermittent Fasting-induced Ischemic Tolerance. Cond. Med..

[25] Park S., Zhang T., Wu X., Qiu J.Y. (2020). Ketone production by ketogenic diet and by intermittent fasting has different effects on the gut microbiota and disease progression in an Alzheimer’s disease rat model. J. Clin. Biochem. Nutr..

[26] Shin B.K., Kang S., Kim D.S., Park S. (2018). Intermittent fasting protects against the deterioration of cognitive function, energy metabolism and dyslipidemia in Alzheime’s disease-induced estrogen deficient rats. Exp. Biol. Med..

[27] Neth B.J., Bauer B.A., Benarroch E.E., Savica R. (2021). The Role of Intermittent Fasting in Parkinson’s Disease. Front. Neurol..

[28] Sokal A., Jarmakiewicz-Czaja S. (2022). Can brain ageing and the risk of dementia be prevented by a proper diet?. Med. Ogólna I Nauk. O Zdrowiu..

[29] Morales-Suarez-Varela M., Collado Sánchez E., Peraita-Costa I., Llopis-Morales A., Soriano J.M. (2021). Intermittent fasting and the possible benefits in obesity, diabetes, and multiple sclerosis: A systematic review of randomized clinical trials. Nutrients.

[30] Bai M., Wang Y., Han R., Xu L., Huang M., Zhao J., Lin Y., Song S., Chen Y. (2020). Intermittent caloric restriction with a modified fasting-mimicking diet ameliorates autoimmunity and promotes recovery in a mouse model of multiple sclerosis. J. Nutr. Biochem..

[31] Hartman A.L., Rubenstein J.E., Kossoff E.H. (2013). Intermittent fasting: A “new” historical strategy for controlling seizures?. Epilepsy Res..

[32] Höhn S., Dozières-Puyravel B., Auvin S. (2019). History of dietary treatment: Guelpa & Marie first report of intermittent fasting for epilepsy in 1911. Epilepsy Behav..

[33] Fann D.Y.-W., Ng G.Y.Q., Poh L., Arumugam T.V. (2017). Positive effects of intermittent fasting in ischemic stroke. Exp. Gerontol..

[34] Kim J., Kang S.W., Mallilankaraman K., Baik S.H., Lim J.C., Balaganapathy P., She D.T., Lok K.Z., Fann D.Y.-W., Thambiayah U. (2018). Transcriptome analysis reveals intermittent fasting-induced genetic changes in ischemic stroke. Hum. Mol. Genet..

[35] Fann D.Y.-W., Santro T., Manzanero S., Widiapradja A., Cheng Y.-L., Lee S.-Y., Chunduri P., Jo D.-G., Stranahan A.M., Mattson M.P. (2014). Intermittent fasting attenuates inflammasome activity in ischemic stroke. Exp. Neurol..

[36] Cox N., Gibas S., Salisbury M., Gomer J., Gibas K. (2019). Ketogenic diets potentially reverse Type II diabetes and ameliorate clinical depression: A case study. Diabetes Metab. Syndr. Clin. Res. Rev..

[37] Hussin N., Shahar S., Teng N.I.M.F., Ngah W.Z.W., Das S.K. (2013). Efficacy of Fasting and Calorie Restriction (FCR) on mood and depression among ageing men. J. Nutr. Health Aging.

[38] Jahrami H.A., Bahammam A.S., Haji E.A., Bragazzi N.L., Rakha I., Alsabbagh A., Nugraha B., Pasiakos S.M. (2021). Ramadan Fasting Improves Body Composition without Exacerbating Depression in Males with Diagnosed Major Depressive Disorders. Nutrients.

[39] Warwick J., Slavova S., Bush J., Costich J. (2020). Validation of ICD-10-CM surveillance codes for traumatic brain injury inpatient hospitalizations. Brain Inj..

[40] Lyeth B.G. (2016). Historical Review of the Fluid-Percussion TBI Model. Front. Neurol..

[41] Kayani N.A., Homan S.G., Yun S., Zhu B.-P. (2009). Health and Economic Burden of Traumatic Brain Injury: Missouri, 2001–2005. Public Health Rep..

[42] Carroll C.P., Cochran J.A., Guse C.E., Wang M.C. (2012). Are we underestimating the burden of traumatic brain injury? Surveillance of severe traumatic brain injury using centers for disease control International classification of disease, ninth revision, clinical modification, traumatic brain injury codes. Neurosurgery.

[43] Hinson H.E., Rowell S., Schreiber M. (2015). Clinical evidence of inflammation driving secondary brain injury: A systematic review. J. Trauma Acute Care Surg..

[44] Weiss E.S., Collett A.E., Kaplan M.J., Omert L.A., Leung P.S.P. (2018). 833: Complement inhibition mitigates secondary effects of traumatic brain injury (TBI). Crit. Care Med..

[45] Cordaro M., Impellizzeri D., Paterniti I., Bruschetta G., Siracusa R., De Stefano D., Cuzzocrea S., Esposito E. (2016). Neuroprotective Effects of Co-UltraPEALut on Secondary Inflammatory Process and Autophagy Involved in Traumatic Brain Injury. J. Neurotrauma.

[46] Van Eldik L.J., Roy S.M., Guptill J.T. (2020). First in human studies of MW0Q 6 189WH, a brain penetrant, ant‰ neuroinflammatory, small molecule drug candidate: Phase 1 safety, tolerability, pharmacokinetic, and pharmacodynamic studies in healthy adult volunteers. Alzheimer’s Dement..

[47] Killen M.J., Giorgi-Coll S., Helmy A.E., Hutchinson P.J., Carpenter K.L.H. (2019). Metabolism and inflammation: Implications for traumatic brain injury therapeutics. Expert Rev. Neurother..

[48] Cao S., Li M., Sun Y., Yang W., Liang W. (2022). Intermittent Fasting Enhances Hippocampal Npy Expression to Promote Neurogenesis Following Traumatic Brain Injury. Nutrition.

[49] Rubovitch V., Pharayra A., Har-Even M., Dvir O., Mattson M.P., Pick C.G. (2019). Dietary Energy Restriction Ameliorates Cognitive Impairment in a Mouse Model of Traumatic Brain Injury. J. Mol. Neurosci..

[50] Har-Even M., Rubovitch V., Ratliff W.A., Richmond-Hacham B., Citron B.A., Pick C.G. (2021). Ketogenic Diet as a potential treatment for traumatic brain injury in mice. Sci. Rep..

[51] Bains M., Hall E.D. (2012). Antioxidant therapies in traumatic brain and spinal cord injury. Biochim. Biophys. Acta.

[52] Hubbard W.B., Harwood C.L., Geisler J.G., Vekaria H.J., Sullivan P.G. (2018). Mitochondrial uncoupling prodrug improves tissue sparing, cognitive outcome, and mitochondrial bioenergetics after traumatic brain injury in male mice. J. Neurosci. Res..

[53] Hubbard W.B., Joseph B., Spry M.L., Vekaria H.J., Saatman K., Sullivan P.G. (2019). Acute Mitochondrial Impairment Underlies Prolonged Cellular Dysfunction after Repeated Mild Traumatic Brain Injuries. J. Neurotrauma.

[54] Davis L.M., Pauly J.R., Readnower R.D., Rho J.M., Sullivan P.G. (2008). Fasting is neuroprotective following traumatic brain injury. J. Neurosci. Res..

[55] Davis L.M.H. The Underlying Mechanism(s) of Fasting Induced Neuroprotection after Moderate Traumatic Brain Injury. Ph.D. Thesis.

[56] Greco T., Glenn T.C., Hovda D.A., Prins M.L. (2016). Ketogenic diet decreases oxidative stress and improves mitochondrial respiratory complex activity. J. Cereb. Blood Flow Metab..

[57] Lee J., Duan W., Long J.M., Ingram D.K., Mattson M.P. (2000). Dietary restriction increases the number of newly generated neural cells, and induces BDNF expression, in the dentate gyrus of rats. J. Mol. Neurosci..

[58] Fontán-Lozano Á., Sáez-Cassanelli J.L., Inda M.C., de los Santos-Arteaga M., Sierra-Domínguez S.A., López-Lluch G., Delgado-García J.M., Carrión Á.M. (2007). Caloric Restriction Increases Learning Consolidation and Facilitates Synaptic Plasticity through Mechanisms Dependent on NR2B Subunits of the NMDA Receptor. J. Neurosci..

[59] Qin W. (2006). Neuronal SIRT1 Activation as a Novel Mechanism Underlying the Prevention of Alzheimer Disease Amyloid Neuropathology by Calorie Restriction. J. Biol. Chem..

[60] Kim S.-E., Mori R., Shimokawa I. (2020). Does Calorie Restriction Modulate Inflammaging via FoxO Transcription Factors?. Nutrients.

[61] Srámková V., Berend S., Siklová M., Caspar-Bauguil S., Carayol J., Bonnel S., Marques M., Decaunes P., Kolditz C.I., Dahlman I. (2019). Apolipoprotein M: A novel adipokine decreasing with obesity and upregulated by calorie restriction. Am. J. Clin. Nutr..

[62] Jessberger S., Clark R.E., Broadbent N.J., Clemenson G.D., Consiglio A., Lie D.C., Squire L.R., Gage F.H. (2009). Dentate gyrus-specific knockdown of adult neurogenesis impairs spatial and object recognition memory in adult rats. Learn. Mem..

[63] Burghardt N.S., Park E.H., Hen R., Fenton A.A. (2012). Adult-Born Hippocampal Neurons Promote Cognitive Flexibility in Mice. Hippocampus.

[64] Wang Z.-M., Liu C., Wang Y.-Y., Deng Y.-S., He X.-C., Du H.-Z., Liu C.-M., Teng Z.-Q. (2020). SerpinA3N deficiency deteriorates impairments of learning and memory in mice following hippocampal stab injury. Cell Death Discov..

[65] Lieberwirth C., Pan Y., Liu Y., Zhang Z., Wang Z. (2016). Hippocampal adult neurogenesis: Its regulation and potential role in spatial learning and memory. Brain Res..

[66] Manzanero S., Erion J.R., Santro T., Steyn F.J., Chen C., Arumugam T.V., Stranahan A.M. (2014). Intermittent fasting attenuates increases in neurogenesis after ischemia and reperfusion and improves recovery. J. Cereb. Blood Flow Metab..

[67] Baik S.H., Rajeev V., Fann D.Y., Jo D.G., Arumugam T.V. (2020). Intermittent fasting increases adult hippocampal neurogenesis. Brain Behav..

[68] Lee J., Seroogy K.B., Mattson M.P. (2002). Dietary restriction enhances neurotrophin expression and neurogenesis in the hippocampus of adult mice. J. Neurochem..

[69] Cheng B., Mattson M.P. (1994). NT-3 and BDNF protect CNS neurons against metabolic/excitotoxic insults. Brain Res..

[70] Robel S., Berninger B., Gotz M. (2011). The stem cell potential of glia: Lessons from reactive gliosis. Nat. Rev. Neurosci..

[71] Xiong X.-Y., Liu L., Yang Q.-W. (2016). Functions and mechanisms of microglia/macrophages in neuroinflammation and neurogenesis after stroke. Prog. Neurobiol..

[72] Xu S., Lu J., Shao A., Zhang J.H., Zhang J. (2020). Glial Cells: Role of the Immune Response in Ischemic Stroke. Front. Immunol..

[73] Alam S.I., Jo M.G., Park T.J., Ullah R., Ahmad S., Réhman S.u., Kim M.O. (2021). Quinpirole-Mediated Regulation of Dopamine D2 Receptors Inhibits Glial Cell-Induced Neuroinflammation in Cortex and Striatum after Brain Injury. Biomedicines.

[74] Jha K.A., Pentecost M., Lenin R.R., Klaj L., Elshaer S.L., Gentry J., Russell J.M., Beland A., Reiner A., Jotterand V. (2018). Concentrated Conditioned Media from Adipose Tissue Derived Mesenchymal Stem Cells Mitigates Visual Deficits and Retinal Inflammation Following Mild Traumatic Brain Injury. Int. J. Mol. Sci..

[75] Sofroniew M.V. (2005). Reactive astrocytes in neural repair and protection. Neuroscientist.

[76] Kumar A., Loane D.J. (2012). Neuroinflammation after traumatic brain injury: Opportunities for therapeutic intervention. Brain Behav. Immun..

[77] Tweedie D., Rachmany L., Kim D.S., Rubovitch V., Lehrmann E., Zhang Y., Becker K.G., Perez E.J., Pick C.G., Greig N.H. (2016). Mild traumatic brain injury-induced hippocampal gene expressions: The identification of target cellular processes for drug development. J. Neurosci. Methods.

[78] Afridi R., Tsuda M., Ryu H.R., Suk K. (2022). The Function of Glial Cells in the Neuroinflammatory and Neuroimmunological Responses. Cells.

[79] Meneghini V., Peviani M., Luciani M., Zambonini G., Gritti A. (2021). Delivery Platforms for CRISPR/Cas9 Genome Editing of Glial Cells in the Central Nervous System. Front. Genome Ed..

[80] Loncarevic-Vasiljkovic N., Pesic V., Todorovic S., Popic J., Smiljanic K., Milanovic D., Ruzdijic S., Kanazir S. (2012). Caloric restriction suppresses microglial activation and prevents neuroapoptosis following cortical injury in rats. PLoS ONE.

[81] Radler M., Wright B., Walker F., Hale M., Kent S. (2015). Calorie restriction increases lipopolysaccharide-induced neuropeptide Y immunolabeling and reduces microglial cell area in the arcuate hypothalamic nucleus. Neuroscience.

[82] Michinaga S., Koyama Y. (2021). Pathophysiological Responses and Roles of Astrocytes in Traumatic Brain Injury. Int. J. Mol. Sci..

[83] Loncarevic-Vasiljkovic N., Pesic V., Tanic N., Milanovic D., Popic J., Kanazir S., Ruzdijic S. (2009). Changes in markers of neuronal and glial plasticity after cortical injury induced by food restriction. Exp. Neurol..

[84] Liu Y., Wang R., Zhao Z., Dong W., Zhang X., Chen X., Ma L. (2017). Short-term caloric restriction exerts neuroprotective effects following mild traumatic brain injury by promoting autophagy and inhibiting astrocyte activation. Behav. Brain Res..

[85] Sharma S., Kaur G. (2008). Dietary restriction enhances kainate-induced increase in NCAM while blocking the glial activation in adult rat brain. Neurochem. Res..

[86] John G.R., Lee S.C., Brosnan C.F. (2003). Cytokines: Powerful Regulators of Glial Cell Activation. Neuroscientist.

[87] Bush T.G., Puvanachandra N., Horner C.H., Polito A., Ostenfeld T., Svendsen C.N., Mucke L., Johnson M.H., Sofroniew M.V. (1999). Leukocyte infiltration, neuronal degeneration, and neurite outgrowth after ablation of scar-forming, reactive astrocytes in adult transgenic mice. Neuron.

[88] Voskuhl R.R., Peterson R., Song B., Ao Y., Morales L., Tiwari-Woodruff S.K., Sofroniew M.V. (2009). Reactive Astrocytes Form Scar-Like Perivascular Barriers to Leukocytes during Adaptive Immune Inflammation of the CNS. J. Neurosci..

[89] Stein D.G., Hoffman S.W. (2003). Concepts of CNS plasticity in the context of brain damage and repair. J. Head Trauma Rehabil..

[90] Schoch K.M., Madathil S.K., Saatman K.E. (2012). Genetic manipulation of cell death and neuroplasticity pathways in traumatic brain injury. Neurotherapeutics.

[91] Yu T.-S., Zhang G., Liebl D.J., Kernie S.G. (2008). Traumatic brain injury-induced hippocampal neurogenesis requires activation of early nestin-expressing progenitors. J. Neurosci..

[92] Baecker J., Wartchow K.M., Sehm T., Ghoochani A., Buchfelder M., Kleindienst A. (2020). Treatment with the Neurotrophic Protein S100B Increases Synaptogenesis Following Traumatic Brain Injury. J. Neurotrauma.

[93] Scheff S.W., Price D.A., Hicks R.R., Baldwin S.A., Robinson S.E., Brackney C.K. (2005). Synaptogenesis in the hippocampal CA1 field following traumatic brain injury. J. Neurotrauma.

[94] Zeng Z., Zhang Y., Jiang W., He L., Qu H. (2020). Modulation of autophagy in traumatic brain injury. J. Cell Physiol..

[95] Huang Y., Long X., Tang J., Li X., Zhang X., Luo C., Zhou Y., Zhang P. (2020). The Attenuation of Traumatic Brain Injury via Inhibition of Oxidative Stress and Apoptosis by Tanshinone IIA. Oxid. Med. Cell Longev..

[96] Chen J., Wang H.-C., Luo C., Gao C., Zhang Y., Chen G., Chen W., Chen X.-P., Tao L. (2020). Chd8 Rescued TBI-Induced Neurological Deficits by Suppressing Apoptosis and Autophagy Via Wnt Signaling Pathway. Cell. Mol. Neurobiol..

[97] Taheri S., Karaca Z.C.O., Mehmetbeyolu E., Hamurcu Z., Yilmaz Z., Dal F., È1nar V., Ulutabanca H., Tans1verdi F., Unluhizarci K. (2022). The Role of Apoptosis and Autophagy in the Hypothalamic-Pituitary-Adrenal (HPA) Axis after Traumatic Brain Injury (TBI). Res. Square.

[98] Loncarevic-Vasiljkovic N., Milanovic D., Pesic V., Tesic V., Brkic M., Lazic D., Avramovic V., Kanazir S. (2016). Dietary restriction suppresses apoptotic cell death, promotes Bcl-2 and Bcl-xl mRNA expression and increases the Bcl-2/Bax protein ratio in the rat cortex after cortical injury. Neurochem. Int..

